# The impact of different mechanical file systems with or without adjunctive approaches on disinfection of oval root canals: an *ex vivo* study

**DOI:** 10.1038/s41415-025-8504-y

**Published:** 2025-07-11

**Authors:** Ebthal Hamada, Abeer H. Mahran, Soha Elhady, Eugenio Pedullà, Kavalipurapu Venkata Teja, Mohammed Turky

**Affiliations:** 002568571023377239328 Department of Endodontics, Faculty of Dentistry, Minia University, Minia, Egypt; 929645060003457425298 Department of Endodontics, Faculty of Dentistry, Ain Shams University, Cairo, Egypt; 188718660294842549446 Department of Medical Microbiology and Immunology, Faculty of Medicine, Ain Shams University, Cairo, Egypt; 213546234018016735003 Department of General Surgery and Surgical-Medical Specialties, University of Catania, Catania, Italy; 576528203333804789975 Department of Conservative Dentistry and Endodontics, Mamata Institute of Dental Sciences, Hyderabad, Telangana, India; 789238243718667686209 Department of Endodontics, Faculty of Dentistry, Minia University, Minia, Egypt; Department of Endodontics, Faculty of Dentistry, Sphinx University, Assiut, Egypt

## Abstract

**Background** The available evidence does not conclusively demonstrate the superiority of a specific file system for effectively disinfecting oval root canals. Additionally, it remains unclear whether adjunctive methods can enhance the disinfection of oval canals.

**Objectives** The study aimed to compare bacterial reduction in oval canals using a single-file versus multi-file system, with or without adjunctive disinfection.

**Methods** In total, 100 human maxillary second premolars with oval single-root canals were contaminated with *Enterococcus faecalis* biofilm. Teeth were divided into two groups: Reciproc Blue single-file and Hyflex CM multi-file. Each group had subgroups: saline irrigation, circumferential filing, sodium hypochlorite irrigation and ultrasonic activation. Bacterial sampling was done pre- and post-chemo-mechanical preparation.

**Results** All instruments significantly reduced bacterial count but did not eliminate infection entirely. Hyflex CM showed higher bacterial reduction than Reciproc Blue, regardless of adjunctive approaches (*p* <0.001). Passive ultrasonic irrigation produced the most significant reduction.

**Conclusions** Multi-file systems substantially reduced the bacterial load in the oval root canals compared to single-file systems. Adjunctive disinfection approaches improved the antibacterial effects of the different file systems employed during the basic chemo-mechanical preparation. Circumferential filing and passive ultrasonic agitation were effective adjunctive approaches in maximising the disinfection in oval root canals.

## Key points

- Owing to their irregular geometry, oval root canals pose a challenging anatomy for effective root canal debridement and disinfection.
- A combination of engine-driven and manual instrumentation along with potent disinfecting chemical agents are required to interact seamlessly in reducing the bacterial load in oval root canals.
- Adjunctive approaches like irrigant agitation are able to improve the disinfection process in such intricate anatomies.
- The present study highlights the utility of concerted action of a multi-file rotary system, thorough root canal irrigation, circumferential filing of the buccal and lingual recesses, and ultrasonic agitation in optimising oval canal disinfection.

## Introduction

Microorganisms play an important role in the development of pulp and periapical diseases which can affect endodontic outcomes.^1^ *Enterococcus faecalis* is a facultative gram-positive bacteria, highly resistant to antimicrobial agents, and it can adhere, grow and penetrate deeply into dentinal tubules and resist host defence. Therefore, it is one of the most common species that causes persistent endodontic lesions.^2^ The phenomenon of microorganisms adhering to and growing uniformly on exposed surfaces was previously identified and led to research that showed surface-associated microorganisms, or biofilms, to have a unique phenotype regarding gene transcription and growth rate. These biofilm-producing microorganisms evoke distinct processes for initial surface adhesion, community organisation, ecosystem growth and dissociation.^3^ Additionally, bacteria within a biofilm can endure challenging growth and environmental circumstances.^4^ In order to control such infections, effective root canal therapy depends on eradicating biofilms through chemo-mechanical preparation. However, the complex morphologies of root canals, including their fins, isthmus and irregular cross-sections, present significant confrontations.^5^^,^^6^^,^^7^

There are various types of root canal cross-sections, including round, oval, long oval, flattened and irregular canals. The buccolingual diameter of oval canals is twice the mesiodistal diameter, whereas the buccolingual diameter of long oval canals is two to four times the mesiodistal diameter.^8^^,^^9^^,^^10^Such configurations are challenging to disinfect due to their hard-to-clean and hard-to-shape forms, which create areas that increase the possibility of forming a bacterial reservoir which may later lead to treatment failure.^11^^,^^12^^,^^13^

Mechanical instrumentation of root canals plays an important role in intracanal bacterial reduction.^14^^,^^15^ Historically, practitioners tend to commonly use manual instrumentation in the shaping process of the root canals during endodontic procedures. However, the evolution of nickel-titanium (NiTi), engine-driven files provided many advantages over manual instrumentation, such as more rapid procedures,^16^ more centred preparations^12^ and less apical extrusion of debris.^17^

With the advent of mechanically heat-treated, engine-driven files, prominent improvements in root canal instrumentation were made.^18^ Hyflex CM (HCM) (Coltene Whaledent, Cuyahoga Falls, OH, USA) is a multi-file root canal preparation system to be used in continuous rotation motion.^19^ It is also a heat-treated NiTi instrument, manufactured of controlled memory (CM) wire,^18^ which gives the file superior flexibility^20^ and the ability to adjust superiorly to the canal anatomy, even retaining its form while avoiding procedural errors.^21^

The single-file concept was created to simplify and make root canal preparation faster,^22^ and the reciprocating kinematic provides simultaneously a more secure and effective motion for the engine-driven NiTi instrument compared to continuous clockwise rotating instruments.^23^^,^^24^ This approach reduces the likelihood of the file binding in the root canal, increasing the instrument's lifetime.^25^^,^^26^ Recent studies pointed out that reciprocation movement can also efficiently reduce the microorganisms in the root canal system.^27^^,^^28^

Reciproc Blue (RB) (VDW GmbH, Munich, Germany) is a new version of the original M-wire Reciproc (VDW GmbH, Munich, Germany), manufactured with an innovative heat treatment that led to its bluish colour. This heat treatment tends to increase the instrument's flexibility and cyclic fatigue resistance, and leads to fewer surface defects than the traditional M-wire.^29^ This file has, at room temperature, an R-phase crystallographic arrangement and an S-shaped cross-section, which has been stated as capable of increasing the cutting efficiency.^30^ Although it is a single-file system, it is available in three different tip sizes: 25/0.08 (R25), 40/0.06 (R40) and 50/0.05 (R50).

It has been reported that the concerted actions of multiple instruments could produce a significantly higher intracanal bacterial reduction compared with single-file systems.^31^^,^^32^^,^^33^^,^^34^However, other studies^35^^,^^36^^,^^37^^,^^38^^,^^39^^,^^40^do not corroborate those findings, while showing that there would be no significant difference between multi-file and single-file systems in intracanal bacterial elimination. In addition, prior reports have highlighted the advantages of single-file systems over muti-file systems in disinfecting oval root canals.^19^^,^^41^

Mechanical instrumentation of oval canals with either manual or engine-driven NiTi instruments tends to leave unprepared buccal and lingual extensions or recesses, leading to untouched areas that may harbour necrotic pulp remnants and bacterial biofilms.^42^^,^^43^ It has been suggested that a combination of both rotary instruments and manual instrumentation, such as by using Hedstrom files, may improve root canal preparation.^44^ Additionally, to achieve an improved debridement and cleaning of the root canals, irrigation is an essential part of root canal preparation, complementing the pivotal role of mechanical instrumentation by targeting regions beyond the reach of the instruments;^45^ however, it has been reported that conventional syringe irrigation protocol tends to leave parts of the root canal system covered with smear layers, bacteria and debris.^46^

Therefore, numerous adjuvant additional techniques have been developed. One of these is passive ultrasonic irrigation, which has recently led to a renaissance of the use of ultrasonics during root canal treatment.^47^ Nevertheless, there is still an ongoing debate concerning the effectiveness of ultrasonic irrigation in improving root canal disinfection.

Considering the uncertainties regarding the superiority of a single-file system over a multi-file one in the elimination of bacterial load from oval root canal systems and the lack of a well-established consensus on the effect of the adjunctive methods, the present study aimed to compare the impact of both root canal preparation concepts on the removal of single-species bacteria from oval canals, with and without different disinfection adjunctive approaches.

The null hypothesis to be tested would be that there was no significant difference in bacterial elimination between single- and multi-file systems, even with different disinfection adjunctive approaches.

## Materials and methods

### Ethical approval

The present study was conducted after approval by the Research Ethics Committee of the Faculty of Dentistry, Minia University (meeting no. 78 with registration no. 493 from 29 March 2021).

### Sample size calculation

In order to determine the ideal sample size required to test the null hypothesis, a power analysis was conducted (G*Power 3.1.9.7, Heinrich Hein University, Düsseldorf, Germany). Considering an alpha threshold of 0.05, a beta of 0.2 (power = 80%) and an effect size of 0.349 (which was computed using data from an earlier study),^35^ an overall combined sample of at least 90 specimens in total would be required. To account for sample loss and compensate for the uncertainty of the assumption, the total sample size was increased to 100 samples.

### Samples selection

Based on anatomical matching for teeth morphology, dimensions, and pulp anatomy and volume using 3D imaging (Papaya 3D plus, Genoray, Gyeonggi-do, Korea), a total of 100 freshly extracted, human, mature, single-rooted, sound maxillary second premolars presenting with an oval (buccolingual diameter was at least twice that of the mesiodistal one) and single-root canal system, classified as Vertucci's Type I,^48^ with its apical end coinciding with the root apex, and presenting an equivalent angle of root/root canal curvature of less than ten degrees curvature, as determined by Schneider's method,^49^ and a radius of less than 5 mm, were selected. The teeth were collected from the Faculty of Dentistry at Minia University, Egypt (outpatient university clinic) and included in the study. Teeth with caries, previous restorations (intra-coronal or extra-coronal), root canal treatment, internal or external root resorption, calcified canals, non-negotiable root canals, and root canals with initial diameters larger than size 20 or those presenting with signs of cracks and/or root fractures were excluded and replaced with ones which strictly adhered to the inclusion criteria. Immediately after extraction and removal of the attached soft and hard tissues, the teeth were disinfected with 5.25% sodium hypochlorite solution (NaOCl) (Omez, Phar Omez, Pharaonic Pharmaceuticals, Egypt) for 30 minutes, and subsequently kept in an immersion of 0.1% thymol solution (Formula e Acao, São Paulo, SP, Brazil) for a maximum of one month until the time of use, aligning with the technical specification ISO/TS 11-405. The entire experiment lasted for approximately four months, including teeth collection, storage in the preservative media, the incubation period for biofilm maturation and conducting experimental procedures. The tooth extractions were scheduled for reasons non-related to the present research (either for orthodontic or periodontal reasons).

### Samples preparation

The conventional access cavity was prepared using a sterile round diamond bur (Komet Den Komet Dental, Braseler GmbH & Co. KG, Lemgo, Germany) and refined using a sterile safe-ended tapered carbide fissure bur (Komet Den Komet Dental, Braseler GmbH & Co. KG, Lemgo, Germany) mounted on a high-speed handpiece. Following access cavity preparation, the pulp chamber was irrigated and soaked in 5.25% NaOCl for five minutes. The canal patency was achieved using a sterile stainless-steel K-file ISO size 10 (Dentsply, Maillefer, Ballaigues, Switzerland).

To standardise the teeth length, the buccal cusps were reduced, adjusting the length to an average of 19 mm. The working length measurement was accomplished based on the visual method in which a sterile stainless-steel K-file ISO size 10 was inserted in the root canal until it could be visualised at the entrance of the apical foramen. The final working length measurement had 1 mm subtracted from this previous length. All tooth specimens were instrumented and enlarged up to a sterile stainless-steel K-file ISO size 20 (Dentsply, Maillefer, Ballaigues, Switzerland) under copious irrigation with 5 mL of 5.25% NaOCl for one minute which was inactivated with 1 mL of 10% sodium thiosulfate. The root apex was then sealed with a composite resin (Filtek Z350, 3M, St. Paul, MN, USA) and nail varnish was used to cover the root surface in order to prevent bacterial leakage. The coronal access was sealed with a temporary restoration (Cavit G, 3M ESPE, Neuss, Germany). Subsequently, teeth were stored in distilled water for 24 hours at 37 ^o^C and 100% humidity.

Before contamination, all specimens were placed inside 20 ml falcon tubes containing distilled water and sterilised in an autoclave at 121 ºC for 15 minutes. To confirm the sterilisation of the specimens, each sample was irrigated with 1 mL of sterile saline solution and a culture was taken using three sterile paper points size 15 (Dentsply, Maillefer, Ballaigues, Switzerland), inserted sequentially into the root canals for one minute each after scrapping the canal walls circumferentially using a sterile stainless-steel H-file ISO size 15. The samples were plated onto brain-heart infusion (BHI) agar plates and incubated at 37 ºC for 21 days. For easier handling and identification, the teeth were mounted vertically in acrylic resin and placed in Eppendorf tubes.

### Microbial inoculation

The clinical isolate of *E. faecalis* (American Type Culture Collection 29212) was cultured for 24 hours at 37 ºC in BHI broth (Oxoid CM225). The cell suspension was adjusted to ensure the number of bacteria >10^8^ colony forming units (CFU)/mL with the addition of 1 mL of this broth to another fresh BHI, which resulted in a suspension. Each canal was filled with 10 μL *E. faecalis* suspension using sterile micropipettes and a sterile K-file ISO size 15 (Dentsply, Maillefer, Ballaigues, Switzerland) was used to spread the bacterial suspension up to the working length uniformly, followed by applying sterilised cotton over the canal orifice. The specimens were then incubated in *E. faecalis* suspension (>10^8^ CFU/ml) at 37 ºC for 21 days. The suspension was refreshed every 72 hours. For each specimen, two samplings were performed, one before preparation and another after preparation.^35^

### Bacterial sampling before instrumentation (to ensure uniform bacterial distribution among the different samples)

Following incubation, sterile saline solution was injected into root canals and uniformly distributed along the whole canal length using a sterile K-file ISO size 15 (Dentsply, Maillefer, Ballaigues, Switzerland). Sterile paper points size 20 (Dentsply, Maillefer, Ballaigues, Switzerland) were inserted after scraping canal walls using a sterile H-file ISO size 20 (Dentsply, Maillefer, Ballaigues, Switzerland) inside the root canals, each one for one minute, and then transferred to sterile plastic tubes containing 1 mL of physiological saline solution through sterile tweezers and vortexed for 30 seconds. Aliquots of 20 μL were plated onto BHI agar plates after tenfold serial dilutions in sterile saline solution and incubated for 48 hours at 37 °C.^14^

After root canal infection with *E. faecalis*, the teeth were randomly allocated into four groups (two experimental groups, one positive control group and one negative control group). Each experimental group was further subdivided into four subgroups according to instrumentation techniques and the irrigation protocols used.

### Experimental and control groups subdivision

All selected teeth (n = 100) were numbered and randomly assigned into the following four groups.

#### Experimental group one: RB group

In this group (n = 40), the root canals were instrumented using a single-file system (RB R40) with all instruments autoclaved before instrumentation at 121 °C for 20 minutes. They were sub-divided into four subgroups: RB-A subgroup (n = 10), in which 10 mL of phosphate-buffered saline for five minutes was used as a root canal irrigant; RB-B subgroup (n = 10), in which circumferential filing using H-file was used under 2 mL of saline irrigation for 20 seconds as an adjunctive approach; RB-C subgroup (n = 10), in which 10 mL of 5.25% NaOCl for five minutes was used as a root canal irrigant; and RB-D subgroup (n = 10), in which intermittent ultrasonic activation of 5 mL of 5.25% NaOCl for one minute (three cycles, 20 seconds each) was used as an adjunctive technique.

#### Experimental group two: HCM group

In this group (n = 40), the root canals were prepared using multiple file systems (HCM up to 40/0.04 size with all instruments being autoclaved before instrumentation at 121 °C for 20 minutes) and subdivided into four subgroups: HCM-A subgroup (n = 10) in which 10 mL of phosphate-buffered saline for five minutes was used as a root canal irrigant; HCM-B subgroup (n = 10) in which circumferential filing using H-file was used under 2 mL of saline irrigation for 20 seconds as an adjunctive approach; HCM-C subgroup (n = 10) in which 10 mL of 5.25% NaOCl solution for five minutes was used as a root canal irrigant; and HCM-D subgroup (n = 10) in which intermittent ultrasonic activation of 5 mL of 5.25% NaOCl for one minute (three cycles, 20 seconds each) was used as an adjunctive technique.

#### Positive control group

This group (n = 10) was used to check for bacterial viability throughout the experiment (the root canals were contaminated but not instrumented).

#### Negative control group

This group (n = 10) was used to check for sterility of the procedures (the root canals were instrumented but not contaminated)

### Shaping and cleaning procedures

#### The single-file system group procedures

After adjusting the torque and the speed according to the manufacturer's instructions, a RB R40 (size 40/0.06) instrument was introduced into the canal (already under an irrigated environment) using a slow in-and-out pecking motion without completely withdrawing the file from the canal. Additional brushing motion against the root canal lateral walls was also performed. The crown-down approach was done at ten cycles of reciprocation per second, and after reaching two-thirds of the working length, the instrument would be taken out of the canal, the canal was irrigated using a syringe method, and the instrument cleaned and used again until it reached the pre-established working length. Irrigation was also carried out again once the root canal instrumentation was completed. Each file was used for one canal preparation only.

#### The multi-file system group procedures

Restricting to the manufacturer's instructions, a 25/0.08 size file was used for coronal flaring, followed by 20/0.04 and 25/0.04 instruments until the working length was reached. After that, a 20/0.06 size file was used to shape the canal's middle third, and finally, the instruments 30/0.04 and 40/0.04 were used to the full working length. The canal was irrigated using a syringe method in between instruments and only one set of files was used to shape each canal.

#### Basic syringe irrigation

During the previously described instrumentation procedures, the irrigation steps were performed either with 10 mL saline solution (as in subgroups RB-A and HCM-A) or with 10 mL of 5.25% NaOCl solution, each for five minutes, per canal (as in subgroups RB-C and HCM-C) and accomplished using the conventional syringe irrigation method. A 30-gauge, closed-end, side-vented irrigating needle (Shanghai F S Shanghai Fanta Dental Materials Co. Ltd., China), mounted on a conventional syringe, was inserted up to 1 mm short of the predetermined working length. To avoid binding to the canal walls, the irrigating needle was moved in an up-down direction. Before bacterial samplings, NaOCl was inactivated by 5 mL of 10% sodium thiosulfate.^50^

#### Adjunctive disinfecting approaches - circumferential H-file filing

A sterile stainless-steel H-file ISO size 40 (Dentsply, Maillefer, Ballaigues, Switzerland) was used in a filing motion (as in subgroups RB-B and HCM-B) along the buccal and lingual recesses of the oval canal (three short strokes for each surface) under 2 mL of saline irrigation for 20 seconds.

#### Adjunctive disinfecting approaches - passive ultrasonic irrigation

At the end of root canal preparation, an E1 Irrisonic tip (Helse Dental Technology, São Paulo, SP, Brazil) was used for passive intermittent ultrasonic activation of 5 mL of 5.25% NaOCl for 20 seconds in each cycle of three. The tip corresponded to a size 20/0.01 and was mounted on an ultrasonic unit with a power setting of two (20%). The ultrasonic tip was inserted passively into each root canal in order to minimise the contact of the instrument with the root canal walls and used in canals flooded with the irrigant.^51^ As mentioned before, NaOCl was inactivated by 10% sodium thiosulfate^50^ before samplings.

All the previously described endodontic procedures were performed by a single experienced endodontist.

### Bacterial sampling after instrumentation

Immediately following the final root canal instrumentation, sterile paper points size 40 (Dentsply, Maillefer, Ballaigues, Switzerland) were used for bacterial culture in the same manner as before instrumentation after scrapping canal walls using a sterile H-file ISO size 40 (Dentsply, Maillefer, Ballaigues, Switzerland). The number of colony-forming units per 1 mL was counted. Visible colonies were observed on every plate and expressed as CFU/mL.^14^

### Statistical analysis

Numerical data were evaluated for normality by examining the data distribution and applying the Shapiro-Wilk test. Bacterial count data showed non-parametric distribution and extreme positive skewness. Log transformation of the data were carried out to correct for the skewness. Leven's test showed a violation of variance homogeneity assumption, so a robust one-way ANOVA (analysis of variance) followed by Games-Howell post-hoc test was used for the analysis. The results were reported as mean and standard deviation values. The significant threshold of p <0.05 was established. R statistical analysis software (version 4.1.3 for Windows, Bell Laboratories, Murray Hill, NJ, USA) was used to conduct the statistical analysis.

## Results

**Fig. 1:**
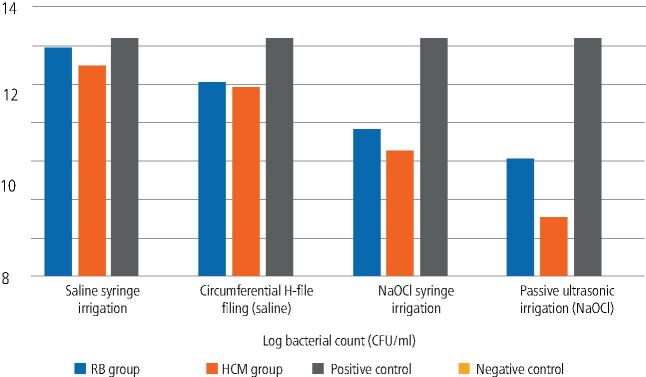
Bar chart showing the average values of bacterial count according to different file systems and supplemental disinfection approaches. It may be observed that the HCM group had always lower CFU counts independently of the disinfection approach, while passive ultrasonic irrigation with NaOCl always had superior disinfection results independently of the file system

The mean and standard deviation values of log bacterial count (CFU/mL) for the different root canal instrumentation systems and adjunctive disinfection approaches are summarised in Table 1 and Figure 1.

**Table 1:** Mean and standard deviation values of bacterial count according to different file systems and adjunctive disinfection approaches

**Adjunctive disinfection approach**	**Log bacterial count (CFU/ml) (mean ± SD)**					
	**RB group**		**HCM group**	**Positive control**	**Negative control**	**p value**
Saline syringe irrigation (A subgroup)	11.90 ± 0.21^c^^,^^3^		10.95 ± 0.36^d^^,^^3^	12.39 ± 0.16^a^	0.00 ± 0.00^b^	<0.001
Circumferential H-file filing (saline) (B subgroup)	10.11 ± 0.63^c^^,^^4^		9.87 ± 0.20^c^^,^^3^			<0.001
NaOCl syringe irrigation (C subgroup)	7.64 ± 0.17^c^^,^^5^		6.58 ± 0.36^d^^,^^4^			<0.001
Passive ultrasonic irrigation (NaOCl) (D subgroup)	6.11 ± 0.25^c^^,^^6^		3.02 ± 0.62^d^^,^^5^			<0.001
Positive control	12.39 ± 0.16^1^					
Negative control	0.00 ± 0.00^2^					
*p* value	<0.001	<0.001				
Superscript letters highlight statistically significant differences among file systems (values within the same line), while superscript numbers highlight statistically significant differences among disinfection approaches (values within the same column)

Regarding the influence of the different file systems, both single-file (RB group) and multi-file (HCM group) systems were able to significantly reduce the bacterial count; although, none of them were able to completely eliminate it under any condition. The HCM group showed significantly lower counts of CFU when compared to RB groups independently of the disinfection approach (p <0.001) (Table 1).

As for the influence of different disinfection approaches, in the RB group, the post-hoc pairwise comparisons revealed statistically significant differences among the different approaches (p <0.001) (Table 1). The passive ultrasonic agitation with NaOCl showed the highest bacterial reduction (lower CFU total counts) (6.11 ± 0.25), followed by conventional syringe NaOCl irrigation (7.64 ± 0.17), and circumferential filing using an H-file (10.11 ± 0.63). The lowest bacterial reduction was observed in saline syringe irrigation (11.90 ± 0.21) (Table 1). In the HCM group, the passive ultrasonic agitation showed the highest bacterial reduction also (3.02 ± 0.62), followed by conventional syringe NaOCl irrigation (6.58 ± 0.36) (p <0.001). The circumferential filing using an H-file and the conventional syringe irrigation with saline subgroups had the lowest bacterial reduction (higher total CFU counts) (9.87 ± 0.20 and 10.95 ± 0.36, respectively) without statistically significant differences between them (Table 1).

## Discussion

Most endodontic failures occur as a result of the presence of bacterial infection;^52^ however, the total elimination of the intra-radicular infection may be a challenge,^53^ especially in complex root canal configurations, such as oval canals.^12^^,^^13^ Root canal preparation file systems appear to be more effective in cross-sectionally rounder root canal systems, a reality that may diverge in oval and long oval root canals,^54^^,^^55^ since the instruments tend to remain centred in their pathway, leaving uninstrumented areas in the recesses of these oval root canals.^54^^,^^56^^,^^57^ These unprepared recesses may often harbour residual bacterial biofilms which may cause persistent infection and consequently, treatment failure.^43^Therefore, supplemental disinfection methods may be required to enhance the role of mechanical instrumentation in intracanal bacterial reduction. The present study aimed to compare the bacterial reduction between two different root canal preparation file systems, a single-file system versus a multi-file system, with and without supplemental disinfection approaches, and concluded that differences exist between both instrumentation groups (RB and HCM groups) and among disinfection protocols also, leading to the rejection of the null hypothesis.

Single-rooted maxillary second premolars with single-root canals were used in the present study as they are one of the most common tooth types presenting with root canal configurations with oval cross-sections.^58^^,^^59^ Mimicking the clinical situation, the access cavity was preferred to decoronation in this *ex vivo* study due to its role as a reservoir of the irrigant, in addition to its effect on root canal procedures.^60^

Despite the diversity of endodontic flora, *E. faecalis* was chosen as it resists the chemo-mechanical preparation, survives even in tough environments, and is one of the most common species found in failed root canal treatments and persistent infection cases. Moreover, it is easy to culture and manipulate.^61^

Although presenting limitations in collecting microorganisms colonising dentinal tubules, ramifications and accessory canals,^62^ the bacteriological sampling was performed through the culture method using paper points as it is an easy and reliable method to detect antibacterial effectiveness.^37^^,^^63^^,^^64^

The present findings suggest that the total elimination of microorganisms from the root canal space is unlikely to happen and that mechanical instrumentation, irrespective of the file system or irrigation used, significantly reduces the bacterial load, which is in agreement with numerous previous studies.^41^^,^^65^^,^^66^^,^^67^

The results of the present study also showed that a multi-file system protocol produced more bacterial reduction compared to a single-file one, regardless of whether a supplemental disinfection approach was used or not. These findings highlight the pivotal role that mechanical instrumentation plays in root canal disinfection^1^^,^^2^ and suggest that the use of more than one root canal preparation file (multiple instruments system) in the shaping procedures might be a relevant factor regarding the maximisation of root canal disinfection, which is an idea that corroborates with previous reports.^31^^,^^32^^,^^33^^,^^34^ However, other studies are not in total agreement with the present findings, since it has been reported that there would be no significant differences between single-file and multiple-file systems in reducing intracanal bacteria.^35^^,^^36^^,^^37^^,^^38^^,^^39^^,^^40^ The difference might be attributed to different samples, instruments, variability in root canal anatomy and different study designs. For instance, a study by Siqueira *et al*.^41^ reported a superior reduction of intracanal infection with a single-file system when compared to a multi-file one. However, this study has used, as a single file, the self-adjusting file, which is a file with specific characteristics (namely the ability to change its form and to deliver continuous irrigation while in service),^56^ which is non-comparable with any other single- or multi-file systems. Notwithstanding these merits, this single-file system was inferior to multi-file systems (BT-Race and ProTaper Next) in reducing the bacterial count in long oval root canals, underscoring the necessity of applying more than one file to combat residual infections in the unprepared buccal and lingual extensions of the root canals,^33^ which aligns with our findings. Prior research has also shown the superiority of a multi-file system (ProTaper Next) over this single-file system.^34^ In addition, Siqueira *et al*. in another study demonstrated that this single-file system possesses a similar disinfecting performance to Reciproc.^68^ Moreover, a previous study underlined the effectiveness of certain expansile single-file systems, like XP-endo Shaper, in achieving better disinfection in oval root canals than the multi-file systems.^19^ Conversely, it has been reported that there was no notable difference in disinfection of oval root canals when XP-endo Shaper was compared to other single-file systems like RB,^69^ while other findings showed that RB outperforms this expansile single file.^70^ Furthermore, a prior study revealed no considerable differences in the disinfecting ability between this expansile single-file system and a multi-file system in infected oval root canals.^40^

The present findings suggest also that circumferential filing of the buccal and lingual recesses of the oval root canals using an H-file is capable of improving mechanical disinfection. This corroborates with Iqbal *et al*.,^44^ and Metzger *et al*.'s^71^ studies. Conversely, Alves *et al*.^37^ did not find any significant additional effect with its use.

The present study confirmed the crucial role irrigation solutions play, besides instruments, in bacterial eradication from the root canal system.^72^ Although the mechanical flushing effect of the irrigation was found to reduce the bacterial count,^73^ Siqueira *et al*.^74^ stated that the irrigant must possess antibacterial properties to maximise canal disinfection. The present study findings showed that indeed, the flushing action of saline solution, which lacks antibacterial properties, delivered using the syringe method, significantly reduced the original bacterial count; however, a superior effect was noted, with conventional syringe NaOCl irrigation, with its powerful antimicrobial activity, showed significantly superior results when compared to saline irrigation in reducing the bacterial intracanal load.

Some controversy exists regarding the role of passive ultrasonic activation of the irrigation solutions in root canal disinfection. The present study showed that passive ultrasonic activation of NaOCl significantly improved bacterial reduction compared to the conventional syringe technique, which is in agreement with previous studies.^46^^,^^75^ Contrary to these findings, other publications^74^^,^^76^^,^^77^exist that were not able to notice any significant difference between both techniques.

The present study finding suggests, therefore, that for maximal disinfection of oval root canals, it is a cumulative action of the use of a multi-file system, circumferential filing of the buccal and lingual untouched (unprepared) recesses, in association with passive ultrasonic activation of NaOCl irrigation solution. The possible reason for this multi-file system superiority, even when using files with lower taper (HCM was used up to 40/0.04 size, while Reciproc R40 has a 40/0.06 size), might be attributed to the insertion of multiple instruments, potentially with multiple insertion angles, which could lead to more interactions with dentin walls and more surface areas touched when comparing to single-file systems, which would tend to require much fewer insertions. Additionally, the total number of irrigation flushes and volume of irrigant delivered in between file insertions might be higher in multiple file systems, which could lead to a superior mechanical flushing effect and mostly to a superior antimicrobial activity when compared to single-file systems. Keeping this in mind, clinicians practising the single-file concept should be attentive and not fall into the temptation of rushing the completion of the root canal treatment, taking advantage of the greater speed of the single-file concept, but investing that extra time in maximising the disinfection protocol which may be harmed when compared to the multi-file system concept.

One of the limitations of the present study is its *ex vivo* nature, which does not allow us to determine a real impact on root canal treatment outcomes. However, although ex vivo studies provide a lower level of evidence, they are a suitable environment for the standardisation of most of the variables that could not be standardised during clinical trials; therefore, the possibility to test and compare different protocols under similar conditions may be seen as a strength of the present research, which simultaneously improves its internal validity. Future well-designed randomised controlled trials are required to compare single- and multi-file systems, with and without supplemental disinfection approaches, while monitoring their influence on the outcome of non-surgical root canal treatment of infected cases.

## Conclusions

Under the conditions of this ex vivo study, it was possible to conclude that all the tested instruments significantly reduced the bacterial load in oval canals but without completely eliminating the infection. The concerted effect of the multi-file system improved the bacterial reduction in oval canals when compared to the single-file system. Circumferential filing of the untouched buccal and lingual areas and passive ultrasonic agitation of NaOCl are required as adjunctive approaches to optimise disinfection in oval root canals.

## Data Availability

All data or materials generated or analysed during this study are included in this article.
